# Behavioral response of *Caenorhabditis elegans* to localized thermal stimuli

**DOI:** 10.1186/1471-2202-14-66

**Published:** 2013-07-03

**Authors:** Aylia Mohammadi, Jarlath Byrne Rodgers, Ippei Kotera, William S Ryu

**Affiliations:** 1 Department of Physics, University of Toronto, Toronto, ON M5S1A7, Canada; 2 Donnelly Centre, University of Toronto, Toronto, ON M5S3E1, Canada; 3 Department of Cell and Systems Biology, University of Toronto, Toronto, ON M5S 3G5, Canada

**Keywords:** Nociception, Thermal sensation, Neuroethology

## Abstract

**Background:**

Nociception evokes a rapid withdrawal behavior designed to protect the animal from potential danger. *C. elegans* performs a reflexive reversal or forward locomotory response when presented with noxious stimuli at the head or tail, respectively. Here, we have developed an assay with precise spatial and temporal control of an infrared laser stimulus that targets one-fifth of the worm’s body and quantifies multiple aspects of the worm’s escape response.

**Results:**

When stimulated at the head, we found that the escape response can be elicited by changes in temperature as small as a fraction of a degree Celsius, and that aspects of the escape behavior such as the response latency and the escape direction change advantageously as the amplitude of the noxious stimulus increases. We have mapped the behavioral receptive field of thermal nociception along the entire body of the worm, and show a midbody avoidance behavior distinct from the head and tail responses. At the midbody, the worm is sensitive to a change in the stimulus location as small as 80 μm. This midbody response is probabilistic, producing either a backward, forward or pause state after the stimulus. The distribution of these states shifts from reverse-biased to forward-biased as the location of the stimulus moves from the middle towards the anterior or posterior of the worm, respectively. We identified PVD as the thermal nociceptor for the midbody response using calcium imaging, genetic ablation and laser ablation. Analyses of mutants suggest the possibility that TRPV channels and glutamate are involved in facilitating the midbody noxious response.

**Conclusion:**

Through high resolution quantitative behavioral analysis, we have comprehensively characterized the *C. elegans* escape response to noxious thermal stimuli applied along its body, and found a novel midbody response. We further identified the nociceptor PVD as required to sense noxious heat at the midbody and can spatially differentiate localized thermal stimuli.

## Background

The ability to sense and react to abrupt, painful changes in the environment is critical for an animal’s survival [1-3]. By evoking reflexive escape behaviors in response to potentially harmful stimuli, organisms are able to avoid possible tissue damage and minimize injury [4,5]. Vertebrates and invertebrates possess sensory neurons called nociceptors that detect noxious stimuli, such as harsh touch or acute heat [6-8]. An animal generally senses these types of stimuli as harmful or potentially damaging, and protects itself with an escape response appropriate to the level of the threat. The nematode *Caenorhabditis elegans* responds to many types of noxious mechanical, osmotic, and chemical stimuli [9-13]. Here we focus on the thermal noxious response of *C. elegans*.

*C. elegans* reacts to noxious temperatures at the head and tail [1,14]. At these extremities, the trajectory of the escape response of a crawling worm is deterministic – if stimulated in the head, the worm will reverse, and if stimulated in the tail, the worm will accelerate forward. Substantial work has been done on the molecular mechanisms of the head and tail noxious responses [1,6,14]. Several neurons have been implicated in the sensation of noxious heat – the FLP and AFD neurons in the head, and the PHC neurons in the tail [6,14]. However, a midbody thermal nociceptor has not yet been identified. In light of the broader spatial receptive field of mechanosensation [4,10] the reported head and tail behavioral responses may be an incomplete characterization of the worm’s ability to respond to thermal noxious stimuli. Therefore, we performed high-content phenotyping of the worm’s thermal noxious response comprehensively along the body of the worm to characterize its spatial dependence.

To perform a systematic quantitative analysis of *C. elegans’* response to localized thermal stimuli, we have developed an assay that allows for the precise spatial and temporal application of an infrared (IR) laser beam to the body of *C. elegans*, and captures its pre- and post-stimulus behavior. We identified key metrics that quantify the response and comprehensively mapped the behavioral receptive field of nociception, revealing a midbody response that is sensitive to very small changes in the stimulus location. Using a multi-dimensional measure of the midbody thermal response behavior, we identified a neuronal candidate (PVD) and a number of molecules (TRPV channels, glutamate) that are involved in the transduction of the nociceptive signals.

## Results

### Novel assay for quantifying the noxious response and mapping the behavioral receptive field

**Figure 1:**
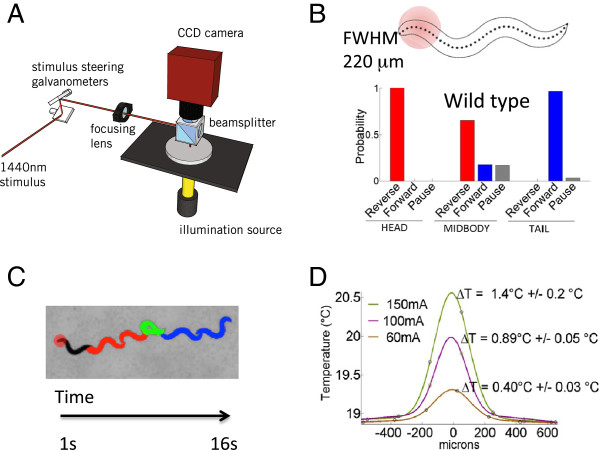
**Assay for the spatial dissection of the thermal noxious response. (A)** Schematic of the localized thermal pulse assay. **(B)** An infrared laser pulse with a 220 um beam diameter (full width half maximum) was used to locally stimulate the entire worm from head to tail, *N* = 442. The head (1–10), midbody (16–26), and tail (31–41) regions are demarcated using the 41 points along the entire “skeleton” of the worm body. An example of the selective targeting of the head is shown. The probability of the first behavioral state after the laser pulse is shown for head, midbody, and tail regions. The head, midbody, and tail responses are statistically different, *p* < 0.001, Fisher’s exact test. **(C)** Raw video data of the worm after a head-applied laser stimulus is shown as a time-lapse sequence. After the laser pulse is applied, the worm enters a reversal (red), followed by an omega turn (green), and then resumes its forward motion (blue). **(D)** Thermal profile of 133 ms IR pulse measured using a thermal camera for three pulse amplitudes (60, 100, and 150 mA), where the largest full width half maximum (FWHM) was measured to be 220 μm for the 150 mA laser pulse.

To date, thermal avoidance assays have been useful in studying the molecular mechanisms of thermal nociception, but have lacked the ability to carefully control variable doses of heat along the body of the worm [2,6,14]. In previous studies where regions along the body of the worm were targeted [1,14], the laser focus was presented for a time long enough (10 seconds) for the heat to diffuse well beyond the worm’s body. Therefore the spatial extent of the stimulus in these experiments is uncertain because the temperature profile in time and space was not clearly shown. In other experiments of thermal nociception, the main drawbacks are that either the whole worm is heated or a thermal barrier selects for sensory neurons in the head [2,6,15]; either case cannot spatially dissect the noxious response. We have addressed these limitations by designing a new thermal avoidance assay that localizes the heat from an IR laser pulse to small regions (approximately 1/5 of the worm’s body length) along the entire body of the worm, and records the behavioral responses to the noxious stimulus (Figure 1a and b). The thermal profile of the beam was carefully calibrated using a thermal camera (Figure 1d, Methods). The beam diameter was measured to be 220 μm (FWHM). This constrained the heating so that the temperature change 500 microns away from the center of the beam is only 2% of the peak, which ensured heating the midbody was not simultaneously heating the head and tail. The temperature of the IR pulse was further independently verified using ratiometric imaging of a temperature sensitive dye pair (rhodamines B and 110) (Methods) [16]. The centroid worm speed and changes in the worm body shape were used to determine the behavioral states of the worm (reverse, forward, omega turn, or pause) before and after the thermal stimuli (Figure 1c, Methods).

### Multi-parameter, high-content phenotyping of N2 noxious response for the head, midbody, and tail

**Figure 2:**
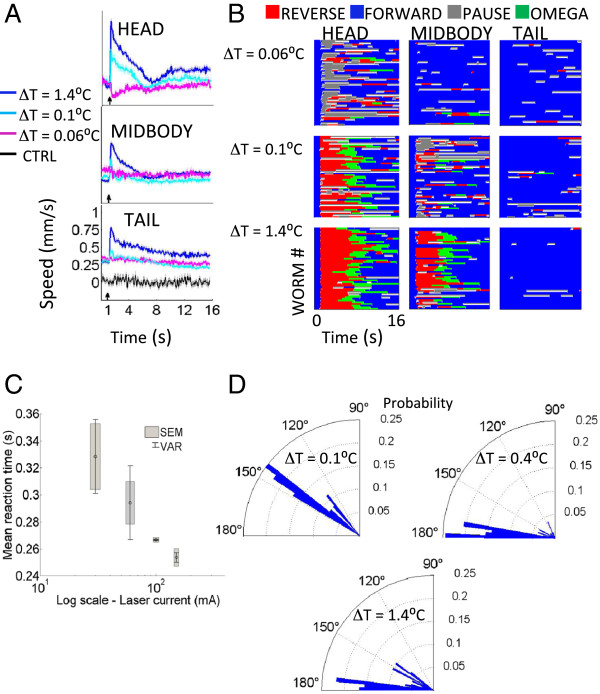
**Spatial high-content phenotyping of N2 thermal noxious response. (A)** The mean centroid speed as a function of time quantifies the dose-dependent differences for the head, midbody and tail responses. Laser is fired at 1 s (arrow). The control speed (CTRL) is offset by 0.25 mm/s for clarity. Shaded regions are SEMs. *N* > 50 for all ∆T > 0°C, *N* = 15 for CTRL. Axes for head and middle are the same as tail. **(B)** Ethograms separated by stimulation region (head, body, tail) for selected ∆T demonstrate the evolution of the behavioral states over time, and show the spatial and thermal variation of the response. **(C)** The reaction time of the worm in response to a head-applied laser stimulus as a function of laser power. **(D)** The probability distribution of the escape angle as the worm responds to a head-applied laser stimulus.

We characterized the reaction of N2 (wild type strain) to a range of temperature ramps along the entire body of the worm to measure the spatial nociceptive dose response. The change in the worm’s centroid speed over time is an informative measure of the thermal response because aspects of this time series change with the stimulus strength. We used this metric to quantify behavioral differences in response to variations in both the power and location of the stimulus (Figure 2a), similar to a previous study [15]. Several features in the dose response scale with power, most notably the maximum mean speed, and the deceleration from the maximum mean speed. The general shape of the mean speed versus time curves also changes in response to the position of the thermal stimulus along the worm body; this is because these speed curves are a product of the underlying locomotory states, some of which change with the position and power of the IR laser. In order to examine these behaviors and further characterize the wild type noxious response, we generated ethograms for the different stimulus laser powers and locations (Figure 2b) [17,18]. At lower laser powers, the behavior is stochastic; as the power increases, the worm’s response becomes more deterministic. From the ethogram, we identified another metric that discriminates the stimuli both by its location and intensity, namely the first behavioral state the worm enters after the stimulus: forward, reverse, and pause. We calculated the probabilities of the first response states (first behavioral states after the stimulus), and measured changes in these probabilities in reaction to changes in the position and power of the stimulus (Figure 1b).

The stereotypical withdrawal response for a crawling *C. elegans* thermally stimulated at the head is a reversal, followed by an omega turn, then a recommencement of forward motion (Figure 1c). The likely purpose of this behavioral series is to make a three-point turn to reorient the worm away from the noxious stimulus. Arguably the worm’s chance to escape danger improves if it is able to respond more quickly to the threat, and reorient itself so that instead of moving towards the hazard it is moving in the opposite direction (180°). We investigated if the escape response improves as a function of the laser power, indicating that these avoidance behaviors changed appropriately for the noxious level of the stimulus. Our results show that the animal’s reaction time does in fact vary inversely with stimulus amplitude (Figure 2c) and that the escape angle increases towards 180° with increasing stimulus power (Figure 2d).

### The noxious response is elicited by a temporal temperature gradient rather than a temperature threshold

Previous studies have used high temperatures in the range of 30°C-35°C to study the noxious response in *C. elegans*[1,2,6,14]. In the context of studying the noxious response, the requirement for high temperature is expected since previous work on mammalian transient receptor potential (TRP) channels in sensory neurons show that a subset of TRPs -- the TRP vanilloid group in particular -- are gated by high temperatures generally > 43°C and have a steep temperature dependence [7,19-21]. Remarkably, our dose response and temperature measurements show that the worm’s robust, stereotypical avoidance response to noxious stimulus at the head can be elicited by relatively small changes in temperature (≤1.4°C) (Figure 1d, Figure 2a). It appears that the temperature ramp rate as opposed to the temperature change above a threshold induces the avoidance response. The ramp rate for the highest temperature stimulus in our dose response is ~9.4°C/s, which is in the noxious range of previous experiments with higher absolute temperatures [15].

**Figure 3:**
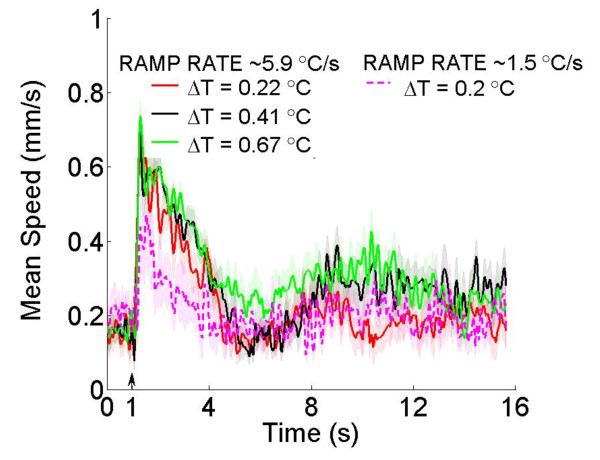
**Withdrawal behavior is dependent on the ramp rate of the thermal stimulus.** The mean speed profile of wild type animals for three head-applied stimuli with the same ramp rate but different absolute temperatures (*N* = 10 for each ∆T), showing a noxious response for each. Animals stimulated with a lower ramp rate but similar ∆T do not exhibit withdrawal behavior, and are statistically different than those stimulated with a higher ramp rate. *p* < 0.05, Kruskal-Wallis test, Dunn’s multiple comparison.

We tested our hypothesis that the ramp rate and not the absolute temperature jump is what produces the thermal nociceptive response by using thermal stimuli with a constant ramp rate but with different ∆Ts (~5.9°C/s, ∆T = 0.22, 0.41, and 0.67°C) (Figure 3). When we stimulated the worm with these short duration, small amplitude thermal pulses, we were able to robustly elicit nearly identical noxious responses (Figure 3). When the ramp rate is lowered and the animal is stimulated with a similar temperature jump (∆T/t ~1.5°C/s, ∆T = 0.2°C), the response is noticeably lower and statistically different compared to the noxious response elicited by the higher ramp rate but same ∆T (Figure 3; Kruskal-Wallis test, Dunn’s multiple comparison *p* < 0.05). This indicates that the avoidance response is dependent on a rate of change in temperature, rather than a crossing of a thermal threshold. Previously reported experiments also stimulated worms with an abrupt change in temperature [1,2,6,14], but our results show that extreme heat is not required to initiate a noxious response if the ∆T/t is above some threshold. For our experiments we stimulated the worm for a fixed duration (133 ms) at different laser powers to produce a range of ∆Ts and ramp rates.

### Spatial sensitivity of the midbody response

**Figure 4:**
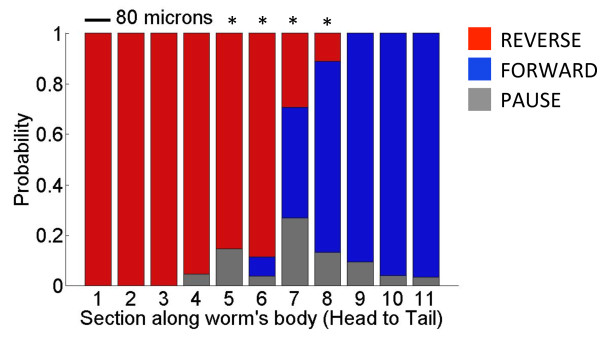
**Spatial sensitivity of the midbody thermal noxious response.** The probability of the first behavioral state after the laser pulse as a function of stimulus location along the worm body is shown. At the midbody the worm is spatially sensitive to changes in beam location as small as 80 microns, and modulates its behavior accordingly. * *p* < 0.05, Fisher’s exact test. *N* = 442 total animals, *N* > 20 each bin.

The midbody behavior is distinct from the head and tail responses (Figure 1b; *p* < 0.001, Fisher’s exact test). At the two extremities of a forward moving worm, the transition to the first state after the stimulus is deterministic—the worm will reverse if stimulated in the head, and will accelerate its forward motion if stimulated in the tail. At the midbody, however, the response is probabilistic as the worm enters a reversal, a forward, or a pause state (Figure 1b). The forward or reverse bias of this behavioral response is strongly correlated with the anterior/posterior position of the stimulus. For example, a laser pulse directed to the anterior middle region closer to the head of the worm will cause a reversal the majority of the time, whereas a laser pulse targeted at the posterior middle has a higher probability to elicit a forward response (Figure 4). We uncovered the “sensory middle” of the worm--a region where the worm may move forward, move backward, or enter a pause state, roughly with equal probability. The brief midbody pause state could arise as a behavioral strategy when there is insufficient asymmetry in the signal, in order to give the worm another opportunity to accrue additional anterior/posterior information about the stimulus before initiating its escape. As the stimulating beam is moved a small distance around this “sensory middle” we can measure a change in the probability of the behavioral response. A statistically significant change in behavior suggests that the worm perceives a difference in the stimuli location. Using this measure we found that statistically the worm has the ability to spatially differentiate the location of our constrained thermal stimuli by as little as 80 microns (Figure 4; *p* < 0.05, Fisher’s exact test).

### Mutant behavioral analyses identify neurons involved in the midbody and tail responses

**Figure 5:**
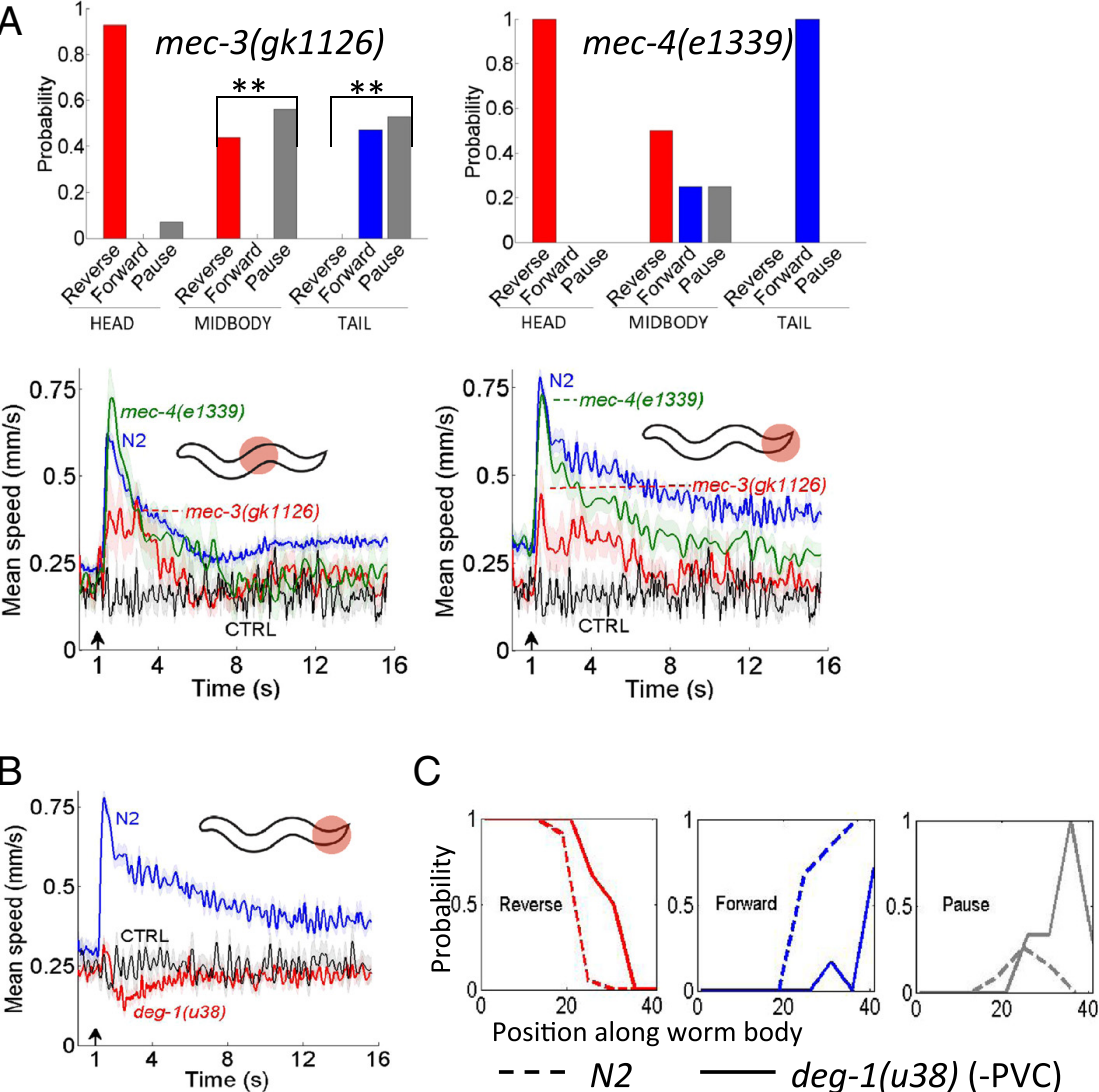
**Strains exhibiting spatially defective thermal avoidance behaviors. (A-B)** The probability of the first behavioral state after the stimulus and the mean speed profiles showing defective behavior compared to N2 for **(A)***mec*-*3*(*gk1126*), ** *p* < 0.01, Fisher’s exact test; behavior compared to N2 for **(A)***mec*-*4*(*e1339*) at the midbody and tail, *p* > 0.05, Fisher’s exact test; defective behavior compared to N2 for **(B)***deg*-*1*(*u38*) at the tail, Kruskal-Wallis test, Dunn’s multiple comparison *p* < 0.0001. *N* = 16 and *N* = 17 for *mec*-*3*(*gk1126*) noxious stimulus at midbody and tail respectively, *N* = 13 *mec*-*3*(*gk1126*) CTRL. *N* = 8 and *N* = 15 for *mec*-*4*(*e1339*) at midbody and tail respectively. *N* = 36 for *deg*-*1*(*u38*) noxious stimulus at tail, *N* = 15 *deg*-*1*(*u38*) CTRL. **(C)** The probability of the first state (Reverse, Forward or Pause) after the stimulus as a function of stimulus location along the body of the worm for N2 and *deg*-*1*(*u38*). *N* = 442 total animals for N2, *N* = 91 total animals for *deg*-*1*(*u38*).

Mutations in the gene *mec-3* affect the development of the bilaterally symmetric pair of nociceptors PVD, such that the neurons lack all but the primary dendritic branching [11,22-24]. We found that *mec-3(gk1126)* had a pronounced defect in the midbody and tail response compared to N2 (Figure 5a; *p* < 0.01, Fisher’s exact test), but the head response showed only a very minor defect (Figure 5a; *p* > 0.05, Fisher’s exact test). PVD has been shown to be the nociceptor for harsh midbody touch [6,10,11], and these results strongly suggest that PVD is also the nociceptor for the midbody thermal avoidance response. Since a mutation in *mec-3* also affects the touch receptor neurons (ALM, AVM, PLM, PVM), we tested the touch resistant *mec-4(e1339)* mutant strain [25] to ensure that the touch neurons were not involved. Our behavioral and speed data show that the *mec-4* mutant response is statistically similar to wild type (Figure 5a; *p* > 0.05, Fisher’s exact test). Since the touch neurons are not involved in transducing the response this leaves PVD as the primary candidate for thermal nociception at the midbody.

Furthermore, PVC has been identified as a command interneuron for the forward tail noxious heat response, being a main synaptic output to the PHC neuron [14]. PVC is also postsynaptic to PVD [26,27]. Our definition of the tail region (Materials and Methods) includes the posterior branching of PVD. Our results show a severe defect in *deg-1(u38)*--a mutant where PVC is degenerated along with four other cell types--in the tail response (Figure 5b; Kruskal-Wallis test, Dunn’s multiple comparison *p* < 0.0001). The tail defect seen in the *mec-3(gk1126)* result (Figure 5a) implicates PVD as a possible nociceptor for the tail response (Figure 5a), suggesting that PVC is acting as the command interneuron in the thermal avoidance circuit in the tail as a postsynaptic target to both PVD and PHC.

PVD chemically synapses equally to AVA (27 synapses) and PVC (28 synapses) [26]. Recent optogenetic analysis of PVD has shown that the probability of backward versus forward movement is determined by the relative synaptic input to the command interneurons as a result of the location of the stimulus along the body of the worm [27]. We investigated this further analyzing *deg-1(u38)* (−PVC) upon noxious stimulation along the body, compared to the wild type response. The loss of functionality of the forward command interneuron effectively shifts the wild type midbody response to the posterior of the worm, and increases the probability of entering a pause state (Figure 5c). This result suggests that the worm’s nociceptive “sensory middle” may be determined by the balance of the synaptic inputs to the command interneurons, and elicits spatially sensitive behavior accordingly (reversal for anterior stimulation, forward for posterior stimulation, and an increased pause state when the signal is symmetric). When the forward command interneuron PVC is not functional, the anterior response (reversal) extends to the posterior, since the PVD-AVA activity dominates.

### PVD is required for the midbody and tail thermal noxious response

**Figure 6:**
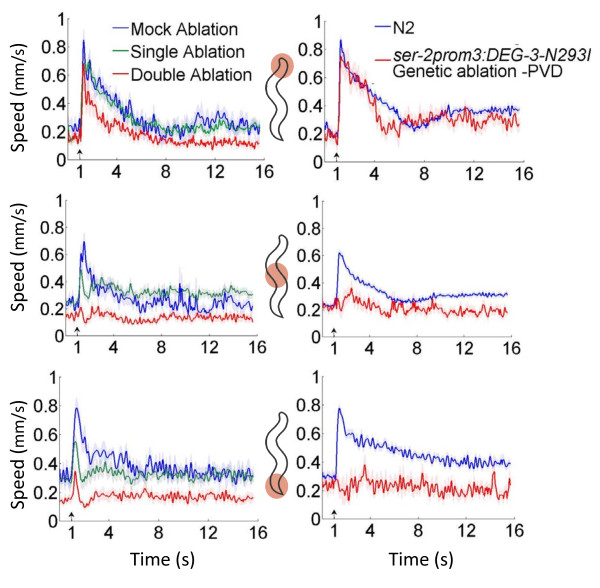
**The PVD sensory neurons mediate the thermal noxious response in the midbody and tail.** Worms with laser ablated PVD neurons (left column) show statistically significant defective thermal avoidance behavior compared to mock ablated worms when stimulated in the midbody and tail (Kruskal-Wallis test, Dunn’s multiple comparison *p* < 0.001); the head response is similar to the mock ablation result. *N* = 9 ablated worms, *N* = 62 total experiments. *ser*-*2prom3*:*DEG*-*3*-*N293I* worms have genetically ablated PVD neurons (right column), and show severe defects in the noxious thermal response for the midbody and tail compared to N2 (Kruskal-Wallis test, Dunn’s multiple comparison *p* < 0.0001). The head response is unaffected. *N* > 12 for each region. Top row: head, middle row: midbody, bottom row: tail.

Our *mec-3* and *mec-4* results suggest that PVD is the sensory neuron underlying the midbody noxious response. In order to confirm its involvement, we eliminated the pair of PVD neurons using laser ablation microsurgery [28]. We generated a transgenic strain expressing cameleon in PVD (Materials and Methods), and ablated both PVDL and PVDR in the late L2 stage. We then tested the response of PVD-ablated young adults to localized thermal stimuli. Our results show the head response is consistent with the mock-ablated response in our transgenic strain, but the midbody and tail responses are severely reduced (Figure 6, left column; Kruskal-Wallis test, Dunn’s multiple comparison *p* < 0.001). We also tested a transgenic strain, *ser-2prom3:DEG-3-N293I*, where PVD is specifically eliminated [24]. These genetic ablation results confirm our laser ablation results – the head response remains unaffected, but we found a clear decrease in the midbody and tail compared to the wild type response (Figure 6, right column; Kruskal-Wallis test, Dunn’s multiple comparison *p* < 0.0001). This demonstrates that the PVD neurons are required for the sensation of noxious heat at the midbody and tail.

*C. elegans* possesses more motor neuron commissures on the right side compared to the left side and accordingly there are more fasciculations with motor neuron commissures from the secondary branches of the PVDR neuron compared to the PVDL neuron [23,26]. This left/right asymmetry led us to investigate the single neuron contribution to the midbody thermal noxious response. Using the same method as the double neuron ablation, we ablated either PVDL or PVDR and tested the head, midbody, and tail responses to our noxious thermal stimulus. The speed versus time analysis suggests that either PVDL or PVDR alone is sufficient since the single neuron ablation data at the midbody and tail respond to the noxious stimulus – although slightly less robustly – compared with mock ablation data (Figure 6, left column).

### PVD responds differently to spatially localized heat pulses targeted at different locations near the midbody

**Figure 7:**
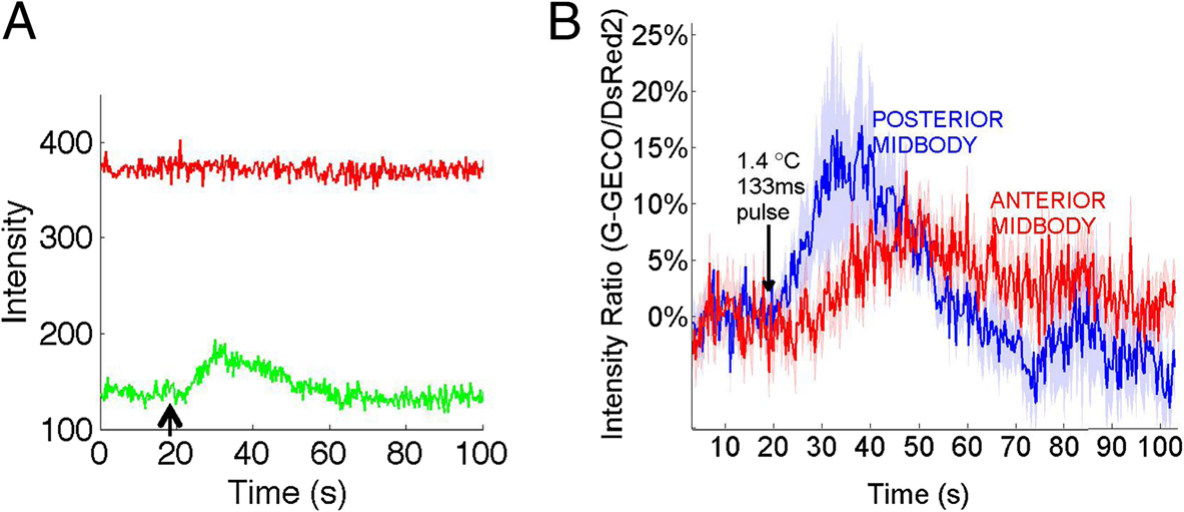
**The PVD neurons show a spatially dependent response to localized noxious heat pulses at the midbody. A)** Sample raw data of G-GECO 1.2 calcium indicator in PVD (green line) and DsRed2 reference chromophore in PVD (red line) during data acquisition, IR pulse at arrow. **B)** Calcium response to IR laser pulse at the midbody for anterior (red line) and posterior (blue line) stimulation. Solid lines represent the average normalized ratio of G-GECO 1.2 emission to DsRed2 emission in PVD for *N* = 8 anterior midbody recordings and *N* = 7 posterior midbody recordings. The arrow is where the IR pulse is applied. Shaded region is SEM.

To show PVD senses localized noxious heat at the midbody, and that it can differentiate the location of the stimuli, we used a G-GECO 1.2 calcium indicator coexpressed with a reference DsRed2 chromophore in the nuclei to measure the influx of calcium into PVD when the midbody is heated with an IR laser pulse at two distinct locations (Figure 7a, 7b). The IR stimulus used for calcium imaging was nearly identical to the one used in the behavior measurements (Materials and Methods). Previous studies applied heat to the whole worm [6,14], which may have selected for a head response and a calcium influx in PVD may not be seen as a result. We measured calcium transients in PVD when stimulated with a 133 ms pulse of heat at two locations, namely anterior to the cell body and posterior to the cell body (Figure 7b). The thermal maxima of the anterior and posterior pulses were approximately 200 μm apart, and fall within the spatially distinct behavioral responses at the midbody that we found in Figure 4. Our calcium transients confirm PVD’s role in transducing the midbody avoidance response. Furthermore, the difference in the signal due to changing the location of the stimulus demonstrates the neuron’s ability to detect a difference between anterior versus posterior stimulation (Figure 7b).

### Mutant strains show defective noxious behavior suggesting molecules involved in sensing heat at the midbody

Our quantitative analysis of 21 mutant strains revealed previously reported results for molecules involved in thermal avoidance for the head and tail [1,14] (Additional file 1: Table S1). Here we focus on those that affect the midbody thermal avoidance response.

**Figure 8:**
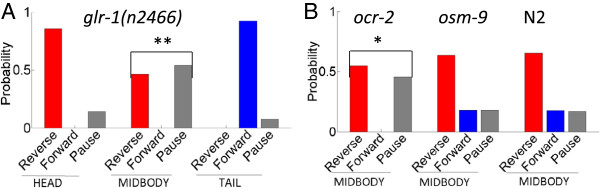
**Potential molecules spatially mediating the thermal avoidance response at the midbody. (A-B)** The probability of the first state after the laser stimulus at the midbody is statistically different compared to N2 for the **(A)***glr*-*1*(*n2466*) mutant strain (** *p* < 0.01, Fisher’s exact test), and **(B)** the *ocr*-*2*(*vs29*) mutant strain (* *p* < 0.05, Fisher’s exact test). The *osm*-*9*(*ky10*) strain shows the same probabilistic midbody response as N2.

The GLR-1 glutamate receptor has been shown to mediate mechanosensory signaling in interneurons postsynaptic to the polymodal nociceptor ASH, and discriminate between sensory inputs [9,29,30]. In particular, *glr-1* mutants are defective to nose touch but not to osmotic shock, even though both modalities are primarily sensed by ASH. GLR-1 is expressed in interneurons controlling locomotion [31], including PVC and AVA which are both direct postsynaptic outputs to PVD. Our results show that a mutation in *glr-1(n2466)* produces a strong midbody behavioral defect (Figure 8a). In particular, the probability of forward locomotion is reduced from 0.18 to 0, the probability of backward motion is reduced from 0.65 to 0.46, and the probability of the pause state dramatically increases from 0.17 to 0.54, relative to the wildtype response. This defective behavior suggests that glutamate could be the transmitter for PVD in the midbody thermal noxious response. This is consistent with the finding that PVD expresses the vesicular glutamate transporter EAT-4, which is required for glutamatergic transmission [32].

The TRPV1 subfamily channels are involved in noxious heat perception in humans and mice [20,21,33]. Recently TRPV channels have been found to contribute to the thermal avoidance response in the head and the tail of *C. elegans*[2,14], and so we investigated their involvement in the midbody thermal noxious response. OCR-2 and OSM-9 are homologues of the mammalian TRPV channel genes in *C. elegans*, and are coexpressed in sensory neurons [34]. Both are expressed in PVD [35-37]. Our results suggest that *ocr-2* is required for noxious heat sensation at the midbody, but *osm-9* is not (Figure 8b). Therefore, it is possible that the OCR-2/OSM-9 heteromer does not function in PVD to control the noxious heat response, as only *ocr-2* produces a behavioral defect.

## Discussion

We developed a high-content thermal nociception assay with precise control of the location of the stimulus in order to spatially dissect the thermal noxious response in *C. elegans*. By reading the “body language” of *C. elegans* as a function of stimulus position, we uncovered a number of new features of thermal nociception, including a midbody response distinct from the known head and tail responses [1,14]. Previous studies [1,2,6,14] used a large change in temperature (>10°C) to elicit escape response, but here we showed that stimuli as small as a fraction of a degree can elicit a response if the change in temperature is fast. In addition the behavioral features of the worm’s response such as the reaction time and the escape angle changes in a way that might be favorable to the worm as the level of the noxious stimuli increases.

Our results also show that the worm can respond to thermal stimuli localized to the midbody. The pair of polymodal nociceptors PVD possess a dendritic arbor that covers most of the worm’s body [23,24], and have been shown to sense aversive stimuli such as harsh touch and cold shock [6,10,11]. Through genetic tools, laser ablation, and calcium imaging, we have confirmed PVD’s involvement in sensing an abrupt increase of heat at the midbody and tail.

Since PVD covers the majority of the worm’s body, it is expected that it would have a large receptive field. Using genetic and neuronal ablation, we were able to delineate the thermally-stimulated receptive field of PVD to the middle and tail regions of the worm. We also generated a behavioral receptive field map for PVD by analyzing the worm’s response to the thermal stimulus as a function of stimulus location along the worm’s body. In doing so, we discovered that *C. elegans* is able to discriminate stimuli at the midbody with a spatial sensitivity of at least 80 microns. This result implies that PVD could be used as a model nociceptor for the study of spatial differentiation of noxious stimuli by a single neuron.

In addition to PVD, we investigated the spatial sensitivity of the midbody response related to the differential synaptic outputs to command interneurons AVA and PVC. It has been recently suggested that relative synaptic inputs to command interneurons due to the position of the stimulus modulate the forward/backward locomotion of the worm [27]. Our measure of the -PVC worm’s behavioral receptive field revealed an extension of AVA initiated reversals into the posterior region of the worm, as well as in increase of pausing in the posterior. In effect, the worm’s “sensory middle” is shifted towards its tail. With PVC not functioning there is no differential excitation of the command interneurons to initiate a spatially biased withdrawal behavior, and the increased pause state in the far posterior may be due to the dominating bias of AVA on the response.

We also discovered a defect in the midbody response of the mutant *glr-1*. Glutamate receptors play an important role in polymodal nociceptors, as they may serve to select for different stimulation modalities [9,29,30]. GLR-1 has also been implicated in long term memory formation [38], as well as the control of locomotion in foraging [31]. Our assay found a defect in the midbody response for the mutant *glr-1(n2466)* (Figure 8a), and this allele is expressed in the command interneurons AVA and PVC [30]. This suggests a role for *glr-1* and glutamate in thermal nociception through PVD.

The utility in quantifying the noxious response goes beyond investigating the midbody thermal avoidance behavior in *C. elegans*. The establishment of *C. eleg*ans as a model organism for nociception requires a comprehensive quantitative analysis of its wild type behaviors to serve as a benchmark in screening for defects caused by genetic, neuronal and pharmacological factors. We generated a dose response and identified several features that scaled with stimulus amplitude and can be used as a measure of nociception. Of note, the maximum mean speed of the response, the probability distribution of the first behavioral state after the stimulus, the reaction time, and the escape angle are all correlated to the strength of the stimulus. Interestingly, we observed that the reaction time is proportional to the logarithm of the stimuli strength, which suggests so-called logarithmic sensing [39] consistent with Weber-Fechner [40] in the sensorimotor transformation for thermal stimuli in *C. elegans*. Regarding the escape angle of the animal, the articulation of the omega turn in the head noxious response modulates the escape trajectory. On average, the omega turn happens several seconds after the stimulus is presented (Figure 2b). Yet, our results show that the worm’s measurement of the strength of the stimulus is incorporated in the omega turn (Figure 2d). In fact, no worms reorient themselves more than 170° when presented with a less harmful stimulus of 30 mA, while 76% of worms do so when the stimulus is more intense (150 mA). In addition the response time decreases and the reversal duration increases as the noxious level of the stimulus increases [15]. Further, the wild type midbody pause state could possibly be explained as a behavioral strategy to allow the worm more time to accrue additional information about the location of the stimulus before breaking the symmetry, and to increase the chances of choosing the trajectory that effectively directs the worm from the danger. *C. elegans* has evolved a complex, multi-faceted behavioral response to a noxious stimulus with multiple features that change in a coordinated way to produce an adaptive protective behavior.

A conceptually similar attempt to quantify pain in animal models is the recent generation of the Mouse Grimace Scale – a catalog of laboratory mouse facial expressions that are meant to quantify the amount of pain felt by acute stimuli [41]. Studies such as these, while promising, have inherent limitations because mammalian behaviors are very complex and difficult to quantify. Furthermore, these animals have a long pre-stimulus history that integrates many environmental stimuli that may confound the pain response. *C. elegans* can be quickly grown in identical conditions and environmental stimuli controlled, making it a desirable model organism for the study of nociception. Furthermore, there is a molecular similarity in thermal nociception or pain among vertebrates and invertebrates [13]. An example of this overlap is the TRPV channel OCR-2 expressed in sensory neurons, which we suggest is required for thermal nociception in PVD. Even though there will be differences in vertebrate and invertebrate nociception, this work adds to the growing evidence that investigating thermal nociception in *C. elegans* may help in understanding this sensorimotor transformation in higher organisms.

## Conclusion

We have developed a novel assay to spatially dissect and quantify the *C. elegans* thermal noxious response with high resolution. The *C. elegans* avoidance response is a multi-faceted behavior where several features change in a way to improve the animal’s escape. Our analysis revealed a spatially sensitive midbody response distinct from the head and tail responses. The nociceptor PVD is required for the sensing of heat at the midbody, and has the ability to spatially differentiate localized stimuli.

## Methods

*C. elegans* strains were grown following standard procedures. Worms were prepared following a previous protocol used for thermal sensory behavioral measurement [15]. Behavior was quantified using custom programs written in LabVIEW and MATLAB as previously published [15]. The thermal stimulus was measured using a thermal IR camera (ICI 7320, Infrared Cameras Inc, TX) and confirmed by ratio dye imaging. Calcium imaging was done using standard techniques with a dual EMCCD Nikon TI (Nikon, USA).

### Strains

Strains were cultivated at 20°C on NGM plates with *E. coli* OP50 according to standard protocols [42]. The strains used in this work were: Wild type N2, *deg-1(u38)*, *flp-21(ok889)*, *glr-1(n2466)*, *mec-3(1338)*, *mec-3(gk1126)*, *mec-4(e1339)*, *mec-10(e1515)*, *mec-10(tm1552*), *npr*-*1*(*ad609*), *npr*-*1*(*ky13*), *npr*-*1*(*n1353*), *ocr*-*2*(*vs29*), *osm*-*6*(*p811*), *osm*-*9*(*ky10*), *sem*-*4*(*n1378*), *tax*-*2*(*p671*), *tax*-*4*(*p678*), *trpa*-*1*(*ok999*), *ttx*-*1*(*p767*), *unc*-*86*(*n846*), obtained from the Caenorhabditis Genomics Center.

We also used *ser*-*2prom3*:*DEG*-*3*-*N293I*, *ser*-*2prom3*:*DEG*-*3*-*N293I*;*mec*-*4*(*e1611*), and *mec*-*10p*:*DEG*-*3*-*N293I*, obtained from the Treinin Lab.

The transgenic strain *akIs11* was obtained from Rajarshi Ghosh, Kruglyak Lab, Princeton University.

### Generation of PVD cameleon line WRP-9

The *ser*-*2* promoter was generated by PCR-amplification from genomic DNA. A plasmid containing *YC3*.*60* was obtained from Mei Zhen, and used to generate *ser*-*2*::*YC3*.*60*. The sequence of the resulting DNA clone was confirmed by sequencing. Transgenic strains were generated by microinjection.

### Generation of pan-neuronal G-GECO/DsRed2 line

We amplified F25B3.3, G-GECO [43], and DsRed2 fragments in separate PCR tubes, and combined them in fusion PCR to generate the F25B3.3::G-GECO-T2A-DsRed2 construct. The plasmid DNA was injected to the distal gonad of N2 strain. We then generated integrated strains by gamma-ray irradiation at 4000 cGy. The integrated strains were back-crossed three times to N2 strain to reduce background mutations.

### Thermal stimulus assay

Worms were assayed in a temperature-controlled room (22.5°C ± 1°C). Images were obtained using a Leica MZ7.5 stereomicroscope and a Basler firewire CMOS camera (A6021-2; Basler, Ahrensburg, Germany). A 2 mm diameter collimated beam through a 100 mm focal length lens from a 1440 nm diode laser (FOL1404QQM; Fitel, Peachtree City, GA) was focused at the surface of the agar, near the center of the camera’s field of view (Figure 1a). The diode laser was driven a Thorlabs controller (LDC 210B and TED 200C; Thorlabs, Newton, NJ). A custom program written in LabVIEW (National Instruments, Austin TX) was used to control the IR laser firing, power, and duration, while simultaneously recording images of the crawling worm at 30 fps for 1 second of pre-stimulus behavior, followed by 15 seconds of post-stimulus behavior. The plate was moved at least 1 second prior to the laser firing so that a random location along the worm’s body was targeted by the laser. The laser and worm positions were simultaneously recorded so the precise location of the pulse when fired is known. The control data for all datasets (examples 0 mA in Figures 2a and 5a,b) show no discernable effect from the careful movement of the plate. Only forward moving worms were assayed, and each worm was stimulated only once. Images were processed offline using custom programs written in MATLAB (Mathworks, Natick, MA). A thermal camera (ICI 7320, Infrared Camera Inc., Texas, USA) was used to measure the temperature of the agar when heated by the IR laser (Figure 1d). The temperature change caused by the 10 mA dose was below the resolution of the thermal camera, so the reported value is from the ratiometric temperature measurement described below. We also measured the thermal profile of the beam with an anesthetized worm at the center, and confirmed that the measurement of the temperature of the agar is the same as the measurement of the worm’s temperature. This indicates that the thermal capacity and conductivity of the worm is nearly identical to that of agar. The noxious stimulus used for the wild type, genetic ablated and mutant analyses was 150 mA. The cameleon strain required a higher dose to elicit the noxious response in the tail, therefore 300 mA was used to assay the ablated and mock ablated animals.

### Rho-B/Rho-110 ratiometric temperature measurement of laser stimulus

An 11 mm diameter cut-out from an agar assay plate was treated with 20 uL of 0.1 mM Rhodamine-B (83695; Sigma) and Rhodamine-110 (R6626; Sigma) in 20 mM HEPES buffer. The relationship between ratio of intensities and temperature for the two-Rhodamine system was calibrated by measuring fluorescent signals from each dye at known temperatures using a thermoelectric cooler and benchtop controller (5R6-900; Oven Industries, Mechanicsburg, PA). Sample temperature was measured using an embedded thermocouple (5TC-TT-T-40-36; Omega, Stamford, CT) and a digital logging thermometer (53II; Fluke, Everett, WA). Imaging was done using an inverted Nikon microscope (Eclipse Ti, Nikon, USA). The sample was excited by a 505 nm LED (M505L1; Thorlabs, Newton, NJ), and emission from each Rhodamine was simultaneously captured by dual EMCCDs (DU-897E-CSO-BV; Andor, Belfast, UK), controlled by NIS-Elements AR (Nikon). The following filter sets were used: (i) 518 nm dichroic (FF518-Di01; Semrock, Rochester, New York) with 494 nm long pass filter (FF01-494/20-25), and (ii) 538 nm dichroic (FF538-FDi01, Semrock) with 531 nm band pass filter (FF02-531/22, Semrock) and 579 nm short pass filter (FF01-579/34, Semrock).

The IR thermal stimulus used in the behavioral assay was replicated and applied to the assay plate. Emission from each Rhodamine was captured at 40.5 FPS, using 2× binning. For each laser current, at least 51 pulses were averaged. The difference in ratio of intensities (Rho-B/Rho-110) between each frame of the time series and the pre-stimulus frame was calculated, and averaged over a 14.5 μm by 14.5 μm (9 by 9 binned pixels) region of interest. The slope of the calibration fit (ΔRatio/ΔTemperature) was used to calculate the change in temperature above baseline at each time-point. Images were processed with custom programs written in MATLAB (Mathworks; Natick, MA). To measure the decay time constant, an exponential fit was made on the decay time course of the laser heating after the peak temperature. These results confirmed our IR camera measurements, and provided temperature values for pulses which were below the resolution of the thermal camera.

### Neuronal ablation

Laser ablation of PVD was performed essentially as previously described, by focusing a 440 nm < 4 ns pulsed dye laser (Duo-220; Laser Science Inc., Franklin, MA) pumped by a nitrogen laser (337205–00, Spectra-Physics, Santa Clara, CA) onto the imaging plane of an inverted microscope (TE-2000E; Nikon) with a NA1.4 100× oil objective (Plan Apo 100X/1.40; Nikon) [44]. Mid-L2 stage *C*. *elegans* expressing *cameleon* in PVD were used to identify the target neurons and align them with the position of the laser beam. The success of each ablation was confirmed using disappearance of YFP fluorescence and the elimination of harsh touch behavioral response. Worms were immobilized for imaging on pads made from 5% or 10% agarose dissolved in M9 buffer with 0.25 μl of 0.1 μm diameter PolyStyrene microspheres (PS02N; Bangs Laboratories, Fishers, IN) 2.5% w/v suspension in M9 buffer. The worms were recovered by transferring the entire pad to an NGM plate with ample food, and releasing the worms from the beads with M9 buffer.

### Calcium imaging

For calcium imaging of PVD’s response to heat at the midbody, we used the same IR thermal stimulus as for the ratiometric Rhodamine B/110 temperature measurement (described above), mounted on top of a Nikon Eclipse Ti microscope with dual EMCCDs (DU-897E-CSO-BV; Andor, Belfast, UK). Optical recordings were obtained from worms expressing a pan-neuronal G-GECO 1.2-based calcium sensor and reference chromophore DsRed2 in the nuclei of the cells. Recordings were done at the cell body, while the IR pulse was focused on the dendrites anterior and posterior to the cell body, the same distance away from the cell body. Neutral density filters were used to limit photobleaching. The following filter sets were used: (i) 495 nm dichroic (T495lpxr; Chroma) with 470 nm bandpass (40 nm) filter (ET470/40×; Chroma), and (ii) 538 nm dichroic (FF538-FDi01, Semrock) with 531 nm bandpass filter (FF02-531/22, Semrock) and 593 nm longpass filter (FF01-593/LP-25; Semrock). Worms were immobilized as they were for neuronal ablation.

## Competing interests

The authors declare no competing financial interests.

## Authors’ contributions

AM and WSR conceived the study and designed the experiments. AM obtained and analyzed the data with suggestions by WSR. AM and JBR performed fluorescence microscopy experiments. IK generated the G-GECO/DsRed2 line. AM and WSR wrote the manuscript. All authors have read and approved the final manuscript.

## Supplementary Material

Additional file 1: Table S1 Mutant strains used for thermal nociception assay. Note: Only two metrics from the behavioral quantification -- max mean speed and standard deviation (MMS ± STD) -- are included in this summary. Units are pixels/frame. *p*^N2^ : p-value compared to N2, Kruskal-Wallis test or Fisher’s exact test performed for all extracted features (ie, including those not shown). Click here for file

## References

[B1] Wittenburg N Baumeister R Thermal avoidance in Caenorhabditis elegans: an approach to the study of nociception Proc Natl Acad Sci USA 1999 96 10477 10482 10.1073/pnas.96.18.10477 10468634 PMC17914

[B2] Glauser DA Chen WC Agin R Macinnis BL Hellman AB Garrity PA Tan M-W Goodman MB Heat avoidance is regulated by transient receptor potential (TRP) channels and a neuropeptide signaling pathway in Caenorhabditis elegans Genetics 2011 188 91 103 10.1534/genetics.111.127100 21368276 PMC3120139

[B3] Maroteaux G Loos M van der Sluis S Koopmans B Aarts E van Gassen K Geurts A Largaespada DA Spruijt BM Stiedl O Smit AB Verhage M High-throughput phenotyping of avoidance learning in mice discriminates different genotypes and identifies a novel gene Genes Brain Behav 2012 11 772 784 10.1111/j.1601-183X.2012.00820.x 22846151 PMC3508728

[B4] Pirri JK Alkema MJ The neuroethology of C. elegans escape Curr Opin Neurobiol 2012 22 187 193 10.1016/j.conb.2011.12.007 22226513 PMC3437330

[B5] Bromm B Treede RD Withdrawal reflex, skin resistance reaction and pain ratings due to electrical stimuli in man Pain 1980 9 339 354 10.1016/0304-3959(80)90048-2 7208079

[B6] Chatzigeorgiou M Yoo S Watson JD Lee W-H Spencer WC Kindt KS Hwang SW Miller DM Treinin M Driscoll M Schafer WR Specific roles for DEG/ENaC and TRP channels in touch and thermosensation in C. elegans nociceptors Nat Neurosci 2010 13 861 868 10.1038/nn.2581 20512132 PMC2975101

[B7] Caterina MJ Schumacher MA Tominaga M Rosen TA Levine JD Julius D The capsaicin receptor: a heat-activated ion channel in the pain pathway Nature 1997 389 816 824 10.1038/39807 9349813

[B8] Xu SY Cang CL Liu XF Peng YQ Ye YZ Zhao ZQ Guo AK Thermal nociception in adult Drosophila: behavioral characterization and the role of the painless gene Genes Brain Behav 2006 5 602 613 10.1111/j.1601-183X.2006.00213.x 17081265

[B9] Kaplan JM Horvitz HR A dual mechanosensory and chemosensory neuron in Caenorhabditis elegans Proc Natl Acad Sci USA 1993 90 2227 2231 10.1073/pnas.90.6.2227 8460126 PMC46059

[B10] Li W Kang L Piggott BJ Feng Z Xu XZS The neural circuits and sensory channels mediating harsh touch sensation in Caenorhabditis elegans Nat Commun 2011 2 315 21587232 10.1038/ncomms1308 PMC3098610

[B11] Way JC Chalfie M The mec-3 gene of Caenorhabditis elegans requires its own product for maintained expression and is expressed in three neuronal cell types Genes Dev 1989 3 1823 1833 10.1101/gad.3.12a.1823 2576011

[B12] Hilliard MA Bargmann CI Bazzicalupo P C. elegans responds to chemical repellents by integrating sensory inputs from the head and the tail Curr Biol 2002 12 730 734 10.1016/S0960-9822(02)00813-8 12007416

[B13] Tobin DM Bargmann CI Invertebrate nociception: behaviors, neurons and molecules J Neurobiol 2004 61 161 174 10.1002/neu.20082 15362159

[B14] Liu S Schulze E Baumeister R Temperature- and touch-sensitive neurons couple CNG and TRPV channel activities to control heat avoidance in Caenorhabditis elegans PLoS One 2012 7 e32360 10.1371/journal.pone.0032360 22448218 PMC3308950

[B15] Ghosh R Mohammadi A Kruglyak L Ryu WS Multiparameter behavioral profiling reveals distinct thermal response regimes in *Caenorhabditis elegans* BMC Biol 2012 10 85 10.1186/1741-7007-10-85 23114012 PMC3520762

[B16] Ebert S Travis K Lincoln B Guck J Fluorescence ratio thermometry in a microfluidic dual-beam laser trap Opt Express 2007 15 15493 15499 10.1364/OE.15.015493 19550834

[B17] Zariwala HA Miller AC Faumont S Lockery SR Step response analysis of thermotaxis in Caenorhabditis elegans J Neurosci 2003 23 4369 4377 12764126 10.1523/JNEUROSCI.23-10-04369.2003 PMC6741103

[B18] Albrecht DR Bargmann CI High-content behavioral analysis of Caenorhabditis elegans in precise spatiotemporal chemical environments Nat Methods 2011 8 599 605 10.1038/nmeth.1630 21666667 PMC3152576

[B19] Voets T Droogmans G Wissenbach U Janssens A Flockerzi V Nilius B The principle of temperature-dependent gating in cold- and heat-sensitive TRP channels Nature 2004 430 748 754 10.1038/nature02732 15306801

[B20] Caterina MJ Rosen TA Tominaga M Brake AJ Julius D A capsaicin-receptor homologue with a high threshold for noxious heat Nature 1999 398 436 441 10.1038/18906 10201375

[B21] Basbaum AI Bautista DM Scherrer G Julius D Cellular and molecular mechanisms of pain Cell 2009 139 267 284 10.1016/j.cell.2009.09.028 19837031 PMC2852643

[B22] Tsalik EL Niacaris T Wenick AS Pau K Avery L Hobert O LIM homeobox gene-dependent expression of biogenic amine receptors in restricted regions of the C. elegans nervous system Dev Biol 2003 263 81 102 10.1016/S0012-1606(03)00447-0 14568548 PMC4445141

[B23] Smith CJ Watson JD Spencer WC O'Brien T Cha B Albeg A Treinin M Miller DM Time-lapse imaging and cell-specific expression profiling reveal dynamic branching and molecular determinants of a multi-dendritic nociceptor in C. elegans Dev Biol 2010 345 18 33 10.1016/j.ydbio.2010.05.502 20537990 PMC2919608

[B24] Albeg A Smith CJ Chatzigeorgiou M Feitelson DG Hall DH Schafer WR Miller DM Treinin M C. elegans multi-dendritic sensory neurons: morphology and function Mol Cell Neurosci 2011 46 308 317 10.1016/j.mcn.2010.10.001 20971193 PMC3018541

[B25] O'Hagan R Chalfie M Goodman MB The MEC-4 DEG/ENaC channel of Caenorhabditis elegans touch receptor neurons transduces mechanical signals Nat Neurosci 2004 8 43 50 15580270 10.1038/nn1362

[B26] White JG Southgate E Thomson JN Brenner S The structure of the nervous system of the nematode Caenorhabditis elegans Philos Trans of the R Soc of Lond. B, Biol Sci 1986 314 1 10.1098/rstb.1986.0056 22462104

[B27] Husson SJ Costa WS Wabnig S Stirman JN Watson JD Spencer WC Akerboom J Looger LL Treinin M Miller DM Lu H Gottschalk A Optogenetic analysis of a nociceptor neuron and network reveals ion channels acting downstream of primary sensors Curr Biol 2012 22 743 752 22483941 10.1016/j.cub.2012.02.066 PMC3350619

[B28] Bargmann CI Avery L Laser killing of cells in Caenorhabditis elegans Methods Cell Biol 1995 48 225 250 8531727 10.1016/s0091-679x(08)61390-4 PMC4442485

[B29] Maricq AV Peckol E Driscoll M Bargmann CI Mechanosensory signalling in C. elegans mediated by the GLR-1 glutamate receptor Nature 1995 378 78 81 10.1038/378078a0 7477293

[B30] Hart AC Sims S Kaplan JM Synaptic code for sensory modalities revealed by C. elegans GLR-1 glutamate receptor Nature 1995 378 82 85 10.1038/378082a0 7477294

[B31] Zheng Y Brockie PJ Mellem JE Madsen DM Maricq AV Euronal control of locomotion in C. elegans is modified by a dominant mutation in the GLR-1 ionotropic glutamate receptor Neuron 1999 24 347 361 10.1016/S0896-6273(00)80849-1 10571229

[B32] Lee RY Sawin ER Chalfie M Horvitz HR Avery L EAT-4, a homolog of a mammalian sodium-dependent inorganic phosphate cotransporter, is necessary for glutamatergic neurotransmission in Caenorhabditis elegans J Neurosci 1999 19 159 167 9870947 10.1523/JNEUROSCI.19-01-00159.1999 PMC3759158

[B33] Caterina MJ Leffler A Malmberg AB Martin WJ Trafton J Petersen-Zeitz KR Koltzenburg M Basbaum AI Julius D Impaired nociception and pain sensation in mice lacking the capsaicin receptor Science 2000 288 306 313 10.1126/science.288.5464.306 10764638

[B34] Tobin DM Madsen DM Kahn-Kirby A Peckol EL Moulder G Barstead R Maricq AV Bargmann CI Combinatorial expression of TRPV channel proteins defines their sensory functions and subcellular localization in C. elegans neurons Neuron 2002 35 307 318 10.1016/S0896-6273(02)00757-2 12160748

[B35] Jose AM Bany IA Chase DL Koelle MR A specific subset of transient receptor potential vanilloid-type channel subunits in Caenorhabditis elegans endocrine cells function as mixed heteromers to promote neurotransmitter release Genetics 2007 175 93 105 17057248 10.1534/genetics.106.065516 PMC1774992

[B36] Colbert HA Smith TL Bargmann CI OSM-9, a novel protein with structural similarity to channels, is required for olfaction, mechanosensation, and olfactory adaptation in Caenorhabditis elegans J Neurosci 1997 17 8259 8269 9334401 10.1523/JNEUROSCI.17-21-08259.1997 PMC6573730

[B37] Kindt KS Viswanath V Macpherson L Quast K Hu H Patapoutian A Schafer WR Caenorhabditis elegans TRPA-1 functions in mechanosensation Nat Neurosci 2007 10 568 577 10.1038/nn1886 17450139

[B38] Rose JK Kaun KR Chen SH Rankin CH GLR-1, a non-NMDA glutamate receptor homolog, is critical for long-term memory in Caenorhabditis elegans J Neurosci 2003 23 9595 9599 14573539 10.1523/JNEUROSCI.23-29-09595.2003 PMC6740458

[B39] Kalinin YV Jiang L Tu Y Wu M Logarithmic sensing in Escherichia coli bacterial chemotaxis Biophys J 2009 96 2439 2448 10.1016/j.bpj.2008.10.027 19289068 PMC2989150

[B40] Thompson RF Foundations of physiological psychology Harper & Row 196723825353

[B41] Langford DJ Bailey AL Chanda ML Clarke SE Drummond TE Echols S Glick S Ingrao J Klassen-Ross T Lacroix-Fralish ML Matsumiya L Sorge RE Sotocinal SG Tabaka JM Wong D van den Maagdenberg AMJM Ferrari MD Craig KD Mogil JS Coding of facial expressions of pain in the laboratory mouse Nat Methods 2010 7 447 449 10.1038/nmeth.1455 20453868

[B42] Brenner S The genetics of Caenorhabditis elegans Genetics 1974 77 71 94 4366476 10.1093/genetics/77.1.71 PMC1213120

[B43] Zhao Y Araki S Wu J Teramoto T Chang YF Nakano M Abdelfattah AS Fujiwara M Ishihara T Nagai T Campbell RE An expanded palette of genetically encoded Ca²^+^ indicators Science 2011 333 1888 1891 10.1126/science.1208592 21903779 PMC3560286

[B44] Fang-Yen C Gabel CV Samuel ADT Bargmann CI Avery L Laser microsurgery in Caenorhabditis elegans Methods Cell Biol 2012 107 177 206 22226524 10.1016/B978-0-12-394620-1.00006-0 PMC3617498

