# Supplementary material for: Behavioral response of Caenorhabditis elegans to localized thermal stimuli
Source: BMC Neurosci. 2013 Jul 3;14:66. doi: 10.1186/1471-2202-14-66 (PMC3703451; doi:10.1186/1471-2202-14-66)
Supplement: Additional file 1: Table S1 — Mutant strains used for thermal nociception assay. Note: Only two metrics from the behavioral quantification -- max mean speed and standard deviation (MMS ± STD) -- are included in this summary. Units are pixels/frame. pN2 : p-value compared to N2, Kruskal-Wallis test or Fisher’s exact test performed for all extracted features (ie, including those not shown). [file 1471-2202-14-66-S1.docx]

**Table S1. Mutant strains used for thermal nociception assay**

Note: Only two metrics from the behavioral quantification -- max mean speed and standard deviation (MMS ± STD) -- are included in this summary. Units are pixels/frame.

*p*^N2^ : p-value compared to N2, Kruskal-Wallis test or Fisher’s exact test performed for all extracted features (ie, including those not shown).

| **Genotype** | **HEAD RESPONSE**  **MMS ± STD *p*^N2^<0.05** | **MIDBODY RESPONSE**  **MMS ± STD *p*^N2^<0.05** | **TAIL RESPONSE**  **MMS ± STD *p*^N2^<0.05** |  |
| --- | --- | --- | --- | --- |

| *akIs11* | 0.54 ± 0.55 | Yes | 0.48 ± 0.70 | Yes | 0.55 ± 0.56 | Yes |
| --- | --- | --- | --- | --- | --- | --- |
| *deg-1(u38)* | 1.57 ± 0.37 | No | 1.18 ± 0.61 | Yes | 0.71 ± 0.40 | Yes |
| *flp-21(ok889)* | 1.71 ± 0.58 | No | 1.49 ± 0.74 | No | 1.59 ± 0.36 | No |
| *glr-1(n2466)* | 1.16 ± 0.59 | Yes | 1.17 ± 0.58 | Yes | 1.44 ± 0.43 | No |
| *mec-3(e1338)* | 1.36 ± 0.38 | No | 1.25 ± 0.62 | Yes | 1.17 ± 0.46 | Yes |
| *mec-3(gk1126)* | 1.23 ± 0.69 | No | 0.86 ± 0.39 | Yes | 0.89 ± 0.67 | Yes |
| *mec-4(e1339)* | 1.54 ± 0.42 | No | 1.45 ± 0.33 | No | 1.46 ± 0.22 | No |
| *mec-10(e1515)* | 1.61 ± 0.54 | No | 1.17 ± 0.68 | Yes | 1.38 ± 0.45 | No |
| *mec-10(tm1552)* | 1.66 ± 0.46 | No | 1.27 ± 0.50 | No | 1.15 ± 0.69 | Yes |
| *N2* | 1.73 ± 0.52 | -- | 1.24 ± 0.62 | -- | 1.56 ± 0.41 | -- |
| *npr-1(ad609)* | 1.42 ± 0.85 | Yes | 1.29 ± 0.61 | Yes | 1.18 ± 0.69 | Yes |
| *npr-1(ky13)* | 1.63 ± 0.64 | No | 1.20 ± 0.60 | Yes | 1.40 ± 0.35 | Yes |
| *npr-1(n1353)* | 1.05 ± 0.62 | Yes | 0.72 ± 0.74 | Yes | 1.23 ± 0.40 | No |
| *ocr-2(vs29)* | 1.39 ± 0.43 | No | 0.91 ± 0.56 | Yes | 0.85 ± 0.42 | Yes |
| *osm-6(p811)* | 1.38 ± 0.59 | Yes | 1.13 ± 0.55 | Yes | 1.30 ± 0.44 | Yes |
| *osm-9(ky10)* | 1.63 ± 0.87 | No | 1.66 ± 0.33 | No | 1.63 ± 0.39 | No |
| *ttx-1(p767)* | 1.17 ± 0.41 | No | 1.48 ± 0.59 | No | 1.24 ± 0.18 | No |
| *sem-4(n1378)* | 1.39 ± 0.92 | No | 0.99 ± 1.21 | Yes | 0.73 ± 0.49 | Yes |
| *tax-2(p671)* | 1.59 ± 0.36 | No | 1.40 ± 0.56 | No | 1.41 ± 0.46 | No |
| *tax-4(p678)* | 1.41 ± 0.54 | No | 1.45 ± 0.80 | No | 1.38 ± 0.45 | No |
| *trpa-1(ok999)* | 1.48 ± 0.23 | No | 1.13 ± 0.71 | No | 1.46 ± 1.07 | No |
| *unc-86(n846)* | 0.92 ± 0.69 | Yes | 0.58 ± 0.51 | Yes | 0.58 ± 0.57 | Yes |
|  |  |  |  |  |  |  |
|  |  |  |  |  |  |  |
|  |  |  |  |  |  |  |
|  |  |  |  |  |  |  |
|  |  |  |  |  |  |  |
|  |  |  |  |  |  |  |
|  |  |  |  |  |  |  |
|  |  |  |  |  |  |  |
